# Epidemiological Survey of Quantitative Ultrasound in Risk Assessment of Falls in Middle-Aged and Elderly People

**DOI:** 10.1371/journal.pone.0071053

**Published:** 2013-08-07

**Authors:** Ling-Chun Ou, Zih-Jie Sun, Yin-Fan Chang, Chin-Sung Chang, Ting-Hsing Chao, Po-Hsiu Kuo, Ruey-Mo Lin, Chih-Hsing Wu

**Affiliations:** 1 Department of Internal Medicine, National Cheng Kung University Hospital Dou-Liou Branch, Yunlin, Taiwan; 2 Department of Family Medicine, National Cheng Kung University Hospital, Tainan, Taiwan; 3 Department of Orthopadics, National Cheng Kung University Hospital Dou-Liou Branch, Yunlin, Taiwan; 4 Institute of Gerontology, National Cheng Kung University Medical College, Tainan, Taiwan; 5 Institute of Behavior Medicine, National Cheng Kung University Medical College, Tainan, Taiwan; 6 Department of Public Health and Institute of Epidemiology and Preventive Medicine, College of Public Health, National Taiwan University, Taipei, Taiwan; Harvard Medical School, United States of America

## Abstract

The risk assessment of falls is important, but still unsatisfactory and time-consuming. Our objective was to assess quantitative ultrasound (QUS) in the risk assessment of falls. Our study was designed as epidemiological cross-sectional study occurring from March 2009 to February 2010 by community survey at a medical center. The participants were collected from systemic sample of 1,200 community-dwelling people (Male/Female = 524/676) 40 years old and over in Yunlin County, Mid-Taiwan. Structural questionnaires including socioeconomic status, living status, smoking and drinking habits, exercise and medical history were completed. Quantitative ultrasound (QUS) at the non-dominant distal radial area (QUS-R) and the left calcaneal area (QUS-C) were measured. The overall prevalence of falls was 19.8%. In men, the independently associated factors for falls were age (OR: 1.04; 95%CI: 1.01∼1.06), fracture history (OR: 1.89; 95%CI: 1.12∼3.19), osteoarthritis history (OR: 3.66; 95%CI: 1.15∼11.64) and speed of sound (OR: 0.99; 95%CI: 0.99∼1.00; p<0.05) by QUS-R. In women, the independently associated factors for falls were current drinking (OR: 3.54; 95%CI: 1.35∼9.31) and broadband ultrasound attenuation (OR: 0.98; 95%CI: 0.97∼0.99; p<0.01) by QUS-C. The cutoffs at -2.5< T-score<-1 derived using QUS-R (OR: 2.85; 95%CI: 1.64∼4.96; p<0.01) in men or T-score ≦-2.5 derived using QUS-C (OR: 2.72; 95%CI: 1.42∼5.21; p<0.01) in women showed an independent association with falls. The lowest T-score derived using either QUS-R or QUS-C was also revealed as an independent factor for falls in both men (OR: 2.13; 95%CI: 1.03∼4.43; p<0.05) and women (OR: 2.36; 95%CI: 1.13∼4.91; p<0.05). Conclusions: Quantitative ultrasounds, measured either at the radial or calcaneal area, are convenient tools by which to assess the risk of falls in middle-aged and elderly people.

## Introduction

It is well known that about 10–40% of community-dwelling people aged 65 year-old or older may have had one episode of falling in one year [1]–[6]. Fall-related injuries, such as joint dislocations, soft tissue injuries and fractures, result in poor quality of life including such things as restricted mobility and functional decline and even result in fatal events [1]–[3]. Around 5–10% of falls cause fractures, and almost 90% of fractures result from falls in older individuals [1], [3], [4], [7]–[9]. As people become more elderly, falls occur more often [3], [5]. Therefore, falls are considered to be a major health hazard in geriatric population [1].

The more risk factors of falls elderly people have, the higher is the prevalence rate of falls [10]. Many risk factors related to falls have been suggested in various studies. Being of older age, being female, polypharmacy, low socioeconomic status, living alone, decreased body mass, decreased bone density, abnormal neuromuscular findings, impaired balance and gait, poor visual function, stroke, and previous falls or fall injuries have been suggested to be associated with falls [1], [5], [6], [10]–[13]. In rehabilitation settings in Australia, the risk of falls has been found to be even higher in men than in women instead [14]. Experts have designed some intervention programs to prevent falls, but the results have been both inconsistent and unsatisfactory [1], [15], [16] with the exception of exercise training in people residing in communities and vitamin D supplementation in populations with low vitamin D levels [1], [17]. It is obvious that there are more risk factors that need to be disclosed. If the high-risk population can be detected earlier, fall prevention will be efficiently practiced.

Bone strength is constituted by both bone density and bone quality [18]–[20]. Some devices can be used to measure bone density or quality, such as DXA or quantitative ultrasound (QUS) [20]–[24]. Lower bone mass measured by DXA in 558 women over 65 years old has been proven to be associated with more risk factors of fall [25]. However, DXA is ionizing radiation, and is more expensive, time-consuming and non-portable. Quantitative ultrasound (QUS) including both the calcaneal and radial areas can be used to evaluate bone quality and screen low bone mass using either broadband under attenuation (BUA) or speed of sound (SOS) [20]–[22], [26], [27]. QUS can be used for screening osteoporosis [20]–[22], [26], [27], and can also be used to predict fractures in both men and women [20], [21], [23], [24]. QUS is radiation-free, portable and more convenient than DXA [20], [26], especially in regard to community surveys. In Switzerland, QUS parameters were used to indirectly predict fall risk in hospitalized elderly women [28]. Of the subjects with T-score higher than −1.5 measured by calcaneal QUS, the fallers had lower T-score than non-fallers [29]. Recently, the gait speed and subsequent falls in 1061 participants that included both men and postmenopausal women (average age: 68±8 years old) were associated with lower SOS as determined using calcaneal QUS [30]. Nevertheless, whether the BUA or SOS could be one of the risk assessments for falls has never been comprehensively studied in an epidemiological survey.

In meta-analysis, the long-term risk of hip fractures in 50 year-old males with previous Colles’ fractures was found to be 8%, which is higher than has been found for those with previous spinal fractures (6%) or with global fractures (3%) [31], [32]. Therefore, the skeletal properties of the forearm may be an indicator of skeletal fragility [31]. In fact, SOS derived using radial QUS has been used to evaluate bone mechanical properties [33] and has been shown to be an independent factor of fracture risk in women [19]. However, which site for QUS measurement is suitable for risk assessment of falls has never been discussed in detail.

As the QUS is a popular tool for both professional and general population, it would have substantial impact to the public health strategy if the QUS can be used for risk assessment of falls, especially in an aging society. The aims of this study were (1) to assess the interrelationships between falls and bone parameters derived using either radial or calcaneal QUS in men and women across a middle-aged and elderly community-dwelling population, and (2) to disclose whether different sites for QUS measurement may play different roles according to gender in risk assessment for falls.

## Materials and Methods

### Study Population

In Taiwan, the elderly population has reached more than 11.0%. Yunlin County, with an elderly population of 15.4%, has been rated as the county with the second highest elderly population in Taiwan [34]. The study subjects were sampled using a systemically stratified sampling method. Two townships of Yunlin County (Douliou and Kukeng) were randomly selected in the first sampling step. Six Lis in Douliou (one Li from each district) and 3 villages in KuKeng were chosen as the following step, according to the proportion of elderly residents in the total population. One in every five houses was selected, and all the residents who satisfied the inclusion criteria were invited to participate in this study. Finally, a total of 1,200 ambulatory subjects (Male/Female = 524/676) aged 40 and older received a study survey at National Cheng Kung University Hospital Douliou Branch from March 2009 to February 2010. The unadjusted response rate was 17.3%. The mean age and sex distribution of respondents were not statistically different from those of non-responders. However, the mean age of the total study subjects (59.4 y/o) was older than the corresponding average age (58.0 y/o) in both Douliou and KuKeng townships in Yunlin County [34]. This study was approved by the Institute Review Board of National Cheng Kung University Hospital (IRB number: ER-98-084). Each subject signed an informed consent before examination.

### Measurements and Questionnaires

After fasting overnight, the anthropometry of the subjects, including the body height and body weight, was measured using DETECTO™. The body mass index (BMI) was calculated accordingly. Subjects were required to complete a structural questionnaire that included disclosure of exercise habits and other lifestyle habits (such as smoking history and alcohol consumption), living and socioeconomic status, past medical conditions, drug history and fracture history. These questionnaires had been validated in previous studies [35]–[37]. A fall was defined as when a community resident met one or more of the following conditions: 1. Subject tilted downward unpredictably when standing up, sitting or walking. 2. Subject unintentionally contacted the floor with his/her body when changing position [35], [38].

We defined subjects to have a habit of moderate exercise if the frequency of exercise was more than 3 times per week, current smoking if participants still smoked 1 pack (20 cigarettes) at least per month for more than 6 months and current drinking if participants still drank 1 time per week for more than 6 months [36]. The total smoking amount (TSA, pack-years) was calculated by the following formula: TSA = [cigarette consumed per day÷20] ×smoking years [36]. The socioeconomic status score was calculated according to a modified Hollingshead’s index of social position and was categorized as high (levels 4 and 5) and low (levels 1–3). According to the literature, patients who have suffered from a cerebrovascular accident (CVA) or who have taken psychiatric drugs will exhibit an increased risk of falls [8]. However, as non-ambulatory CVA subjects were excluded, only 13 elderly stroke subjects with normal activity levels were enrolled as subjects in this study.

Each subject completed the QUS measurement in less than 5 minutes. Their bone parameters, including broadband ultrasound attenuation (BUA, dB/MHz) using left calcaneal quantitative ultrasound (QUS-C, CUBA CLINICAL™, MK2.6, McCue Ultrasonics, Winchester, UK; coefficients of variation: 1.3%), speed of sound (SOS, cm/sec) using non-dominant radial quantitative ultrasound (QUS-R, Sunlight Omnisense 7000SP; coefficients of variation: 2∼4%) and T-scores derived from calcaneal or radial QUS were obtained. In this study, the correlation coefficients between QUS-C or QUS-R and bone mineral density as derived from dual energy X-ray absorptiometry (DXA, GE-Lunar, Prodigy) were 0.510–0.588 and 0.241–0.310, respectively. The QUS-derived T-scores using Asian references were used for the classification as normal (T-score ≧−1.0), low bone mass (osteopenia) (−1.0< T-score<−2.5) and osteoporosis (T-score ≦ −2.5) according to the definition available from the World Health Organization [18].

### Data Analyses and Statistical Methods

Data were collected and analyzed using the Statistical Package of Social Science for Windows software Version 16 (SPSSWIN, version 16.0, Chicago, USA). All the continuous variables, such as age, body mass index, total smoking amount, SOS derived using radial QUS and BUA derived using calcaneal QUS were expressed as means (SD). The categorical variables including socioeconomic status, living alone, drinking, moderate exercise, past history of HTN, DM, arrhythmia, OA, psychiatric drug use and fractures were all expressed as percentages. Subjects were divided into either a faller group or a non-faller group. The comparisons between fallers and non-fallers in regard to categorical variables were analyzed using Chi-square test, and continuous variables were analyzed using independent *t* test. Logistic regression models were used to evaluate the independent associations of risk factors for falls by gender. The BUA and SOS of the QUS, either in regard to continuous or categorical T-scores, were used for risk assessment of falls in different logistic regression models. Statistical significance was defined as p<0.05 for two-tailed analysis.

## Results

In Table 1, the overall prevalence rate of falls was 19.8% in subjects aged over 40 years old. Females (20.7%) had a relatively higher prevalence of falls than did males (18.7%), but without statistical significance (p = 0.442). Fallers were older than non-fallers in both male or female subjects. Compared with non-fallers, more current alcohol drinking in female fallers, but higher prevalence of fracture history, DM history and OA history in male fallers were found. Female fallers showed statistically significant lower BUAs in calcaneal QUS, and faller females and males were both characterized by lower SOSs in radial QUS, either according to continuous values, T-scores or lowest T-scores.

**Table 1:** Basic characteristics between faller and non-faller men and women.

Variables		Men (N = 524)			Women (N = 676)		
		Faller (%)	Non-Faller (%)		Faller (%)	Non Faller (%)	
Case number		98 (18.7)	426 (81.3)		140 (20.7)	536 (79.3)	
Age (y/o), mean(SD)		66.4 (10.5)^b^	60.3(11.6)		60.1 (11.2)^b^	57.3(10.6)	
Body mass index (kg/m^2^), mean(SD)		24.59(3.29)	25.58(3.36)		24.46(3.91)	24.33(3.64)	
Low social economic status (Hollingshead’sindex <4)		66 (67.3)	261 (61.3)		102(72.9)	384(71.6)	
Living alone		7 (7.1)	20 (4.7)		11(7.9)	38 (7.1)	
Current smoker	18 (18.4)		91 (21.4)	3 (2.1)		9 (1.7)	
Total smoking amount (pack-years), mean (SD)		11.08 (23.39)	12.11 (23.46)		0.56 (4.08)	0.50 (3.93)	
Current alcohol drinking		21 (21.4)	100 (23.5)		8 (5.7)^a^	13 (2.4)	
Moderate exercise habit		21 (24.9)	106 (21.4)		25 (17.9)	102 (19.0)	
Fracture history		31 (31.6)^a^	92 (21.6)		32 (22.9)	86 (16.0)	
Diabetes Mellitus history		16 (16.3)^a^	37 (8.7)		16 (11.4)	45 (8.4)	
Hypertension history		35 (35.7)	117 (27.5)		28 (20.0)	117 (21.8)	
Arrhythmia history		7 (7.1)	17 (4.0)		6 (4.3)	23 (4.3)	
Osteoarthritis history		6 (6.1)^a^	8 (1.9)		9 (6.4)	33 (6.2)	
Psychiatric drug use		6 (6.1)	12 (2.8)		11 (7.9)	36 (6.7)	
Radial QUS, SOS (cm/s), mean (SD)		3940.33(121.29)^b^	3994.59(166.50)		3940.31(184.22)^a^	3981.39(169.42)	
Radial QUS by T-score							
T-score≧−1		23 (23.5)^b^	199 (46.7)		33 (23.6)^b^	169(31.5)	
−1>T-score>−2.5		55 (56.1)^b^	151 (35.4)		41 (29.3)^b^	190(35.4)	
T-score ≦−2.5		20 (20.4)^b^	76 (17.9)		66(47.1)^b^	177(33.1)	
Calcaneal QUS, BUA (dB/MHz), mean (SD)		70.30(17.17)	70.74(15.77)		60.16(18.06)^b^	66.03(16.22)	
Calcaneal QUS by T-score							
T-score≧−1		37 (37.8)	183 (43.0)		33 (23.5)^b^	168(31.3)	
−1>T-score>−2.5		51 (52.0)	215 (50.5)		67 (47.9)^b^	301 (56.2)	
T-score ≦−2.5		10 (10.2)	28 (6.5)		40 (28.6)^b^	67 (12.5)	
Lowest T-score by radial or calcaneal QUS							
T-score≧−1		10 (10.2)^a^	94 (22.1)		13 (9.3)^b^	89 (16.6)	
−1>T-score>−2.5		62 (63.3)	236 (55.4)		48 (34.3)^b^	241 (45.0)	
T-score ≦−2.5		26 (26.5)	96 (22.5)		79 (56.4)^b^	206 (38.4)	

Comparisons between faller and non-faller.

a p<0.05,

b p<0.01, QUS = quantitative ultrasound, BUA = broadband of ultrasound attenuation, SOS = speed of sound.

To disclose whether different sites of QUS measurement may play a different role by gender in risk assessment of falls, different parameters for radial and calcaneal QUS were used in the logistic regression model for both genders. In the regression model using radial QUS, the SOS (as either a continuous (OR: 0.99; [95%CI: 0.99∼1.00]; p<0.05) or a categorical variable (OR: 2.85; [95%CI: 1.64∼4.96]; p<0.01) was an independently negative risk factor for falls in males only. Age, fracture history and osteoarthritis history were the remaining independent risk factors in males, as were the age and current alcohol drinking factors in females (Table 2). In the regression model using calcaneal QUS, the BUA (as either a continuous (OR: 0.98; [95%CI: 0.97∼0.99]; p<0.01) or a categorical variable (OR: 2.72; [95%CI: 1.42∼5.21]; p<0.01) was an independently negative risk factor for falls in females only. Age, fracture history and osteoarthritis history were the remaining independent risk factors in males, as was current alcohol drinking in females (Table 3).

**Table 2:** Logistic regression models of associated factors, including radial quantitative ultrasound (QUS), for falls in men and women.

Variables	Men (N = 524)		Women (N = 676)		
	Model I	Model II	Model I	Model II	
	OR (95% CI)	OR (95% CI)	OR (95% CI)	OR (95% CI)	
Age (y/o)	1.04 (1.01 to 1.06)^b^	1.04 (1.02 to 1.06)^b^	1.03 (1.00 to 1.05)^a^	1.02 (1.00 to 1.05)^a^	
Body mass index (kg/m^2^)	0.97 (0.90 to 1.04)	0.96 (0.89 to 1.04)	1.01 (0.96 to 1.07)	1.01 (0.96 to 1.07)	
Social economic status (Hollingshead’sindex ≧ 4 = 0, <4 = 1)	1.25 (0.74 to 2.11)	1.24 (0.73 to 2.11)	0.89 (0.57 to 1.39)	0.89 (0.57 to 1.39)	
Living alone (alone = 1, lives with others = 0)	1.24 (0.46 to 3.39)	1.22 (0.45 to 3.37)	0.89 (0.43 to 1.84)	0.86 (0.41 to 1.79)	
Total smoking amount (pack-years)	1.00 (0.99 to 1.00)	1.00 (0.99 to 1.01)	1.00 (0.96 to 1.05)	1.00 (0.96 to 1.05)	
Current alcohol drinking(Yes = 1, No = 0)	1.14 (0.64 to 2.03)	1.12 (0.62 to 2.00)	3.53 (1.35 to 9.21)^a^	3.37 (1.29 to 8.82)^a^	
Moderate exercise habit(Yes = 1, No = 0)	0.76 (0.44 to 1.31)	0.79 (0.46 to 1.37)	1.04 (0.68 to 1.61)	1.07 (0.69 to 1.65)	
Fracture history(Yes = 1, No = 0)	1.89 (1.12 to 3.19)^a^	1.85 (1.09 to 3.14)^a^	1.43 (0.89 to 2.29)	1.43 (0.89 to2.30)	
Diabetes Mellitus history (Yes = 1, No = 0)	1.52 (0.75 to 3.05)	1.61 (0.80 to 3.26)	1.38 (0.73 to 2.60)	1.41 (0.75 to 2.66)	
Hypertension history (Yes = 1, No = 0)	1.13 (0.67 to 1.89)	1.11 (0.66 to 1.88)	0.71 (0.43 to 1.17)	0.71 (0.43 to 1.18)	
Arrhythmia history(Yes = 1, No = 0)	1.57 (0.57 to 4.31)	1.57 (0.56 to 4.46)	0.95 (0.36 to 2.47)	0.92 (0.35 to 2.40	
Osteoarthritis history(Yes = 1, No = 0)	3.66 (1.15 to 11.64)^a^	3.73 (1.14 to 12.13)^a^	0.83 (0.37 to 1.85)	0.82 (0.37 to 1.82)	
Psychiatric drug use(Yes = 1, No = 0)	2.62 (0.84 to 8.15)	2.53(0.81 to 7.92)	1.16 (0.56 to 2.41)	1.14 (0.55 to 2.37)	
Radial QUS, SOS (cm/sec)	0.99 (0.99 to 1.00)^a^	–	0.99 (0.99 to 1.01)	–	
SOS, T-score ≧−1		1.00		1.00	
SOS, −1>T-score>−2.5		2.85 (1.64 to 4.96)^b^		0.99 (0.59 to 1.69)	
SOS, T-score ≦−2.5		1.75 (0.87 to 3.52)		1.52 (0.88 to 2.63)	

Model I: SOS by radial QUS as continuous variable. Model II: categorical T-score by radial QUS.

a p<0.05,

b p<0.01, SOS = speed of sound.

**Table 3:** Logistic regression models of associated factors, including calcaneal quantitative ultrasound (QUS), for falls in men and women.

Variables	Men (N = 524)		Women (N = 676)		
	Model I	Model II	Model I	Model II	
	OR (95% CI)	OR (95% CI)	OR (95% CI)	OR (95% CI)	
Age (y/o)	1.05 (1.02 to 1.07)^b^	1.04 (1.02 to 1.07)^b^	1.02 (0.99 to 1.04)	1.02(0.99 to 1.04)	
Body mass index (kg/m^2^)	0.97 (0.90 to 1.04)	0.97 (0.90 to 1.05)	1.02 (0.97 to 1.08)	1.02 (0.97 to 1.08)	
Social economic status (Hollingshead’s index≧ 4 = 0, <4 = 1)	1.25 (0.74 to 2.10)	1.22 (0.72 to 2.05)	0.88 (0.56 to 1.38)	0.84 (0.54 to 1.33)	
Living alone (alone = 1, lives with others = 0)	1.24 (0.46 to 3.34)	1.18 (0.44 to 3.16)	0.83 (0.40 to 1.74)	0.81 (0.38 to 1.71)	
Total smoking amount (pack-years)	1.00 (0.99 to 1.01)	1.00 (0.99 to 1.01)	1.00 (0.95 to 1.05)	1.00 (0.95 to 1.05)	
Current alcohol drinking(Yes = 1, No = 0)	1.05 (0.59 to1.86)	1.07 (0.60 to 1.90)	3.54 (1.35 to 9.31)^a^	3.36 (1.28 to 8.80)^a^	
Moderate exercise habit(Yes = 1, No = 0)	0.74 (0.43 to 1.27)	0.74 (0.43 to 1.27)	1.09 (0.70 to 1.68)	1.08 (0.70 to 1.67)	
Fracture history(Yes = 1, No = 0)	1.87 (1.11 to 3.15)^a^	1.86 (1.11 to 3.12)^a^	1.44 (0.90 to 2.30)	1.51 (0.94 to 2.43)	
Diabetes Mellitus history (Yes = 1, No = 0)	1.63 (0.81 to 3.24)	1.60 (0.80 to 3.19)	1.41 (0.74 to 2.66)	1.43 (0.75 to 2.73)	
Hypertension history (Yes = 1, No = 0)	1.15 (0.69 to 1.93)	1.15 (0.69 to 1.92)	0.73 (0.44 to 1.20)	0.74 (0.44 to 1.23)	
Arrhythmia history(Yes = 1, No = 0)	1.61 (0.60 to 4.36)	1.61 (0.59 to 4.38)	0.91 (0.35 to 2.37)	0.86 (0.32 to 2.27)	
Osteoarthritis history(Yes = 1, No = 0)	3.70 (1.18 to 11.63)^a^	3.53 (1.11 to 11.23)^a^	0.85 (0.38 to 1.90)	0.80 (0.35 to 1.80)	
Psychiatric drug use(Yes = 1, No = 0)	2.76 (0.91 to 8.41)	2.81 (0.92 to 8.59)	1.21 (0.58 to 2.53)	1.28 (0.61 to 2.68)	
Calcaneal QUS, BUA (dB/MHz)	1.01 (0.99 to 1.02)	–	0.98 (0.97 to 0.99)^b^	–	
BUA, T-score ≧−1		1.00		1.00	
BUA, −1>T-score>−2.5		1.01 (0.61 to 1.65)		1.05 (0.63 to 1.72)	
BUA, T-score ≦−2.5		1.21 (0.50 to 2.92)		2.72 (1.42 to 5.21)^b^	

Model I: BUA by calcaneal QUS as continuous variable. Model II: categorical T-score by calcaneal QUS.

a p<0.05,

b p<0.01, BUA = broadband of ultrasound attenuation.

Because discordant T-scores between calcaneal and radial QUS were not uncommon, the lowest T-score derived from either calcaneal or radial QUS was used as the predictor for the further analysis in the logistic regression model for falls. With the exception of the independent factors of age, current alcohol drinking, fracture history and osteoarthritis history, the osteopenic group (−1.0< T-score<−2.5) showed a significantly higher risk of falls (OR: 2.13; [95%CI: 1.03∼4.43]; p<0.05) as compared to the normal group (T-score ≧−1.0) in males, and the osteoporotic group (T-score ≦−2.5) showed a significantly higher risk of falls (OR: 2.36; [95%CI: 1.13∼4.91]; p<0.05) as compared to the normal group (T-score ≧−1.0) in females (Table 4).

**Table 4:** Logistic regression models of associated factors, including the lowest T-score derived by either calcaneal or radial quantitative ultrasound, for falls in men and women.

Variables	Men (N = 524)	Women (N = 676)
	OR (95% CI)	OR (95% CI)
Age (y/o)	1.04 (1.02 to 1.07)^b^	1.02 (0.99 to1.04)
Body mass index (kg/m^2^)	0.97 (0.90 to 1.05)	1.01 (0.96 to 1.07)
Social economic status (Hollingshead’s index≧ 4 = 0, <4 = 1)	1.25 (0.74 to 2.11)	0.90 (0.57 to 1.41)
Living alone (alone = 1, lives with others = 0)	1.15 (0.43 to 3.09)	0.84 (0.40 to 1.75)
Total smoking amount (pack-years)	1.00 (0.99 to 1.01)	1.00 (0.95 to 1.05)
Current alcohol drinking(Yes = 1, No = 0)	1.06 (0.60 to 1.90)	3.45 (1.31 to 9.11)^a^
Moderate exercise habit(Yes = 1, No = 0)	0.76 (0.44 to 1.31)	1.09 (0.70 to 1.68)
Fracture history(Yes = 1, No = 0)	1.90 (1.13 to 3.20)^a^	1.45 (0.90 to 2.32)
Diabetes Mellitus history (Yes = 1, No = 0)	1.60 (0.80 to 3.20)	1.40 (0.74 to 2.66)
Hypertension history (Yes = 1, No = 0)	1.15 (0.69 to 1.93)	0.73 (0.44 to 1.20)
Arrhythmia history(Yes = 1, No = 0)	1.57 (0.56 to 4.35)	0.88 (0.33 to 2.30)
Osteoarthritis history(Yes = 1, No = 0)	3.50 (1.11 to 11.05)^a^	0.82 (0.37 to 1.82)
Psychiatric drug use(Yes = 1, No = 0)	2.82 (0.91 to 8.70)	1.11 (0.53 to 2.32)
T-score≧−1	1.00	1.00
−1>T-score>−2.5	2.13 (1.03 to 4.43)^a^	1.32 (0.67 to 2.63)
T-score ≦−2.5	1.72 (0.75 to 3.92)	2.36 (1.13 to 4.91)^a^

a p<0.05,

b p<0.01.

## Discussion

Consistent with previous reports in Taiwan [35] and worldwide [2]–[5], the prevalence of falls was 19.8% in middle-aged and elderly people living in the community in this study. The falls occurring outdoors (n = 151) were more than those occurring indoors (n = 87) in this study. This finding was compatible with previous community-based studies [35], [39], [40]. Although the frequency of falls occurring outdoors or indoors might be different [39], [40], the impact of falls is viewed as unanimously universal and is considered a major health hazard of an aging society.

Considering the conventional risk factors of falls [1], only age, alcohol drinking, fracture history and osteoarthritis history were significantly associated with falls. It is not surprising to find the falls to increase along with age [1]. The habits of smoking and alcohol drinking have also been suggested to influence health status that is high risk of frailty, and then increased the risk of falls [41]. Because 5–10% of falls cause a fracture, and almost 90% of fractures result from falls in older individuals [8], [9], a history of fractures may also reflect vulnerability to fall injuries and increase the risk of falling [1]. In our study, a history of osteoarthritis was found to be associated with a risk of falls. Studies have reported that subjects with knee OA report a higher prevalence of falls during the year previous to these studies [39], [42], which is higher than that of the general population. According to the literature, self-reported symptoms of arthritis or foot problems are positively associated with falls, and lower limb muscle strength is also positively associated with falls [5]. Therefore, patients complaining of foot problems or arthritis symptoms may suffer from postural instability or gait problems, which then results in falls [5]. Compatible with the latest reviews [1], a history of diabetes, hypertension, psychiatric drug use or arrhythmia show increasing trends in regard to falls, but these factors did not reach statistical significance after adjustment for all other risk factors in this study.

Physical performance, including gait speed and stability, has been assessed as indicators of frailty [41], . It has been well confirmed that frailty is positively correlated with falls [41], [44]. Although there is no statistical significance after multivariate adjustment, a consistent trend of lower risk of falls in subjects with moderate exercise habits may reflect the importance of physical performance in risk assessment of falls. On the other hand, low gait speed is significantly and positively correlated with bone status as measured using QUS in community dwelling postmenopausal women [30]. Quadriceps muscle strength has also been positively correlated with BUA as derived using calcaneal QUS [42]. In our study, the SOS and BUA were significantly associated with risk of falls even after adjustment with regard to exercise habits. Relatively speaking, the measurement of gait speed and stability was more time-consuming and risky of injuries than QUS. Therefore, QUS parameters may be another surrogate of physical performance by which to assess the risk of falls in middle -aged and elderly people.

Based on bone architecture and the pathophysiology of bone fragility in both women and men, cortical bone loss to be more predominant in middle-aged men and loss of connectivity of trabecular bone has been shown to be predominant in adult women [45], [46]. In men aged 50 and over, previous Colles’ fractures have been shown to create a higher risk of hip fractures as compared to previous spinal fractures [31], [32]. In addition, according to bone structure, there is sexual difference in the rate of radial bone loss in the middle-aged population [18]. For the purpose of measuring bone content, calcaneal QUS has been used in areas with more trabecular bone but thinner cortical bone, and radial QUS has been typically used for measuring cortical bone [26], [27], [33]. In this study, the BUA derived from calcaneal QUS in women and SOS derived from radial QUS in men showed discrepant but independent association with falls. The findings were compatible with the gender-specific pathophysiology of bone loss [31], [46] and thus are convenient for risk assessment of falls in clinical practice and community surveys.

There are several limitations in this study that should be of concern. Those who are frail or non-ambulatory may have a higher risk of falls during daily activities [41]. However, because fall prevention focused on ambulatory subjects tends to be more practical as compared to that conducted on bed ridden people [17], we didn’t enroll people who were handicapped or bed ridden. In this study, only the radial and calcaneal QUS were used, and it is uncertain if the findings can be applied to many other brand or type of QUS. Muscle power, stability and physical performance were not measured concomitantly. According to research conducted in Australia, heel quantitative ultrasound is positively correlated with the muscle power and the stability of the lower extremities [47]. After adjustment for exercise level, QUS is still an independent risk factor of falls in this study. Therefore, it is plausible to conclude our findings across the confounding effects of physical performance. Finally, this is a cross-sectional study on aging society. Despite the relative low response rate, the 1200 study subjects were quite large enough to satisfy the statistical analysis for interrelationships between QUS and falls. However, the causal relationship will be evidenced with long-term cohort study. Of course, any extrapolation to different regions should be undertaken with caution and warrants further study.

### Conclusions

Calcaneal QUS in women and radial QUS in men can be an effective screening tool for risk assessment of falls in community dwelling middle-aged and elderly people.
